# Sensitive and high throughput quantification of abscisic acid based on quantitative real time immuno-PCR

**DOI:** 10.1186/s13007-018-0371-y

**Published:** 2018-11-24

**Authors:** Yi Su, Wei Li, Zhigang Huang, Ruozhong Wang, Weigui Luo, Qing Liu, Jianhua Tong, Langtao Xiao

**Affiliations:** 1 Hunan Provincial Key Laboratory of Phytohormones and Growth Development, Hunan Agricultural University, Changsha, China; 2 Southern Regional Collaborative Innovation Center for Grain and Oil Crops in China, Hunan Agricultural University, Changsha, China; 3 Tea Research Institute, Hunan Academy of Agriculture Science, Changsha, 410125 China

**Keywords:** qIPCR, ABA, Biotin, Avidin

## Abstract

**Background:**

Abscisic acid (ABA) functions as a stress phytohormone in many growth and developmental processes in plants. The ultra-sensitive determination of ABA would help to better understand its vital roles and action mechanisms.

**Results:**

We report a new sensitive and high throughput quantitative real time immuno-PCR (qIPCR) method based on biotin–avidin linkage system for ABA determination in plants. ABA monoclonal antibody (McAb) coated on the inner surface of PCR well pretreated with glutaraldehyde. The pre-prepared probe complex, including biotinylated McAb, biotinylated DNA and streptavidin linker, was convenient for high throughput operations. Finally, probe DNA was quantified by real-time PCR. The detectable ranges were from 10 to 40 ng/L with a limit of detection (LOD) of 2.5 fg. ABA contents in plant sample were simultaneously analyzed using LC–MS/MS to validate the qIPCR method. The results showed that qIPCR method has good specificity and repeatability with a recovery rate of 96.9%.

**Conclusion:**

The qIPCR method is highly sensitive for ABA quantification for actual plant samples with an advantage of using crude extracts instead of intensively purified samples.

## Background

The phytohormone abscisic acid (ABA) is a sesquiterpenoid which plays important functions on many bio-processes in plants [1–3]. As the basic supporting technology for action mechanism study, highly sensitive ABA quantification in trace plant sample is therefore eagerly required by present intensive ABA studies because of its increasing importance in plant science and agriculture. Before 1970s, the epidermal strip stomatal opening test has been employed as the bioassay according to ABA-mediated inhibition of the light-induced stomata opening [4, 5]. Afterwards, enzyme linked immunosorbent assay (ELISA) has been employed to quantify several plant growth substances including ABA since the late 1960s [6]. Commercial phytohormonal ELISA kits are still available today [7, 8], and the limit of detection (LOD) is around the nanogram leve [9, 10]. Radioimmunoassay (RIA) has been employed to quantify ABA with better reproducibility [11, 12]. However, specific Liquid Scintillation Counter (LSC) is needed to measure radioactivity and an official license for researchers is usually required to deal with isotopes in RIA. Along with the progress in instrumental technology, gas chromatography (GC) [13], high performance liquid chromatography (HPLC) [14], gas chromatography–mass spectrometry (GC–MS) [15] and liquid chromatography–mass spectrometry (LC–MS) [16] have successively contributed to ABA quantification. At present, the most popular and widely recognized quantification method for ABA is tandem mass spectrometry because of its high sensitivity [17–19]. In addition, several electrochemical sensors have been developed to quickly quantify ABA according to the electrochemical properties of ABA molecule [10, 20, 21]. Phytohormone-inducible promoter-reporter systems such as pAtHB6T::LUC and ProRAB18::GFP have been constructed and applied in ABA quantification to sketchily reveal its distribution in plant tissues [22, 23]. Although the existing quantification methods can supply some choices for phytohormonal quantification, highly sensitive tandem mass method needs complicated operation and high costs, and the sensitivities of other available methods are usually limited. Therefore, new method in ABA quantification both with easy operation and high sensitivity is still urgently needed.

Immuno-polymerase chain reaction (IPCR) has been studied for quantification since 1990s [24]. The method can rapidly and specifically magnify the detectable signals of analytes, thus allows highly efficient quantification in samples containing target molecules at low concentration since it integrates the advantages of both PCR and immunoassay [25]. PCR has been proved to be a highly sensitive, specific and efficient technique for DNA detection and theoretically capable to sense a single nucleotide [26]. ELISA based on antibodies allows analysis for a broad range of organic molecules, but it has much lower sensitivity compared to PCR. Thus, IPCR has been established by integrating PCR and ELISA.

IPCR is performed in various modes depending on the aim of the experiment. Similar to ELISA, the main reaction manners include the direct, indirect, sandwich, indirect sandwich and competitive [27–30]. Using PCR as the signal amplification system, the sensitivity of IPCR was significantly enhanced comparing with ELISA [31]. Along with the technical development, the introduction of quantitative real time PCR (QRT-PCR) instruments enables IPCR to perform high throughput analysis. IPCR including qIPCR were widely utilized in medical and environmental fields for the quantification of organic-molecule/organism, such as antibodies, proteins, toxins, nucleic acids and pathogens [25, 27, 32, 33]. Regretfully, no report has been presented in phytohormone quantification through IPCR. Therefore, we developed a qIPCR method using biotin–avidin system for sensitive and high throughput ABA quantification in trace plant sample. After strategy design and condition optimization, this ABA qIPCR could simultaneously perform accurate and high throughput analysis of ABA even in a 96-well plate. The limit of detection (LOD) could reach 2.5 fg, to the best of our knowledge, a sensitivity close to that of LC–MS/MS.

## Materials and methods

### Reagents and buffers

ABA monoclonal antibody (McAb) was provided with a titer of 1:1000 and showed fine specificity in our previous work [34]. Biotinylation primer and McAb were performed by Sangon Biotech (Shanghai, China). DNA polymerase and TransStart Green qPCR SuperMix kit was ordered from TransGen Biotech (Beijing, China). ^2^H-ABA was purchased from Olchemim Ltd. (Olomouc, Czech Republic). Bovine serum albumin (BSA) and streptavidin were ordered from Sigma-Aldrich Co. LLC (USA). Analytical-grade glutaraldehyde was purchased from Sangon Biotech (Shanghai, China). Agarose gel DNA extraction kit was purchased from Dingguo Biotech (Beijing, China). Washing buffers included TBS (tris-buffered saline, pH 7.5), PBS (phosphate-buffered saline, pH 7.4), and PBST (phosphate-buffered saline plus Tween, pH 7.5). Coating buffer was 50 mmol/L carbonate buffer (pH 9.5).

### Amplification and purification of biotinylated probe DNA

Biotinylated double-stranded DNA (250 bp) with no homologous sequence in plant was generated by PCR amplification using pUC19 as the template through forward primer (biotin-5′-TATGCAGTGCTGCCATAACCATGA-3′) and reverse primer (5′-ATTGTTGCCGGGAAGCTAGAGTAAGTAGTT-3′). The reaction mixture, in a total volume of 100 μL, contained 10 μL 10 × PCR buffer, 2 μL dNTP (5 μmol/L), 2 μL primer (10 μmol/L), 2.5 U Taq polymerase, and 10 pg template pUC DNA. PCR conditions were 4 min at 94 °C followed by 30 cycles of 30 s at 94 °C, 30 s at 55 °C, and 20 s at 72 °C. The PCR product labeled with biotin was analyzed on 1.5% agarose gel stained with ethidium bromide and then purified by an agarose gel DNA extraction kit. To obtain highly purified biotinylated DNA, HPLC purification were employed.

### PCR tube/plate preparation and ABA monoclonal antibody coating

To confirm the type of PCR tube, the homogeneity of PCR tube/plate was analyzed. Certain volume of water was pipetted into different type of polypropylene (PP) PCR tube/plate including normal PCR tube, normal 8-strip tube, QRT-PCR tube and 96-well QRT-PCR plate. The homogeneity of PCR tube/plate was determined through observing the waterline height. Then, the inner surface of PCR tube/plate was modified with glutaraldehyde. 100 µL 0.8% glutaraldehyde was piped into PCR tube/plate and incubated for 2 h at room temperature. Then after, the solution was removed and the PCR tube/plate was washed three times with PBS. Monoclonal antibody (10 μL 20 μg/mL) against ABA was added in the bottom of PCR plate and incubated for 2 h at 4 °C. Then, the McAb solution in PCR tube/plate was removed and the tube/plate was washed three times with washing buffer. About 100 μL 1% bovine serum albumin (BSA) was added to each well and followed by incubation for 60 min at 4 °C to block the residual adsorption sites and 5 times of washing with PBS. For the coating efficiency calculation, the protein in the removed McAb solution were detected by using Bio-Rad Protein Assay Dye Reagent Concentrate (Cat No. 500-0006) according to the protocols in the user manual.

### Probe complex preparation

The probe complex was consisted of biotinylated McAb, biotinylated DNA and streptavidin linker. A certain rate of three components was mixed in PBS. After incubating for 2 h at 4 °C, the probe complex solution was ultra-filtrated at 5000 g for 15 min at 4 °C through 100 kD ultra-filter tube (EMD Millipore UFC910024) to remove the unlinked probe DNA. The ultra-filter was washed three times using PBS through centrifugation at 5000*g* for 15 min at 4 °C and then the solution in ultra-filter tube was pipetted into another 2 mL centrifugal tube and stored at − 20 °C. Moreover, the amount of linked/unlinked probe DNA was quantified by Eppendorf BioPhotometer plus for the linking efficiency evaluation.

### Binding kinetics analysis of biotin-McAb and biotin-DNA with avidin

One fmol avidin and different mass (1–4 fmol) of biotin-McAb and biotin-DNA was mixed in 50 µL PBS, then incubated for 2 h at 4 °C. The mix was ultra-filtrated at 5000 g for 15 min at 4 °C through 100 kD ultra-filter tube (EMD Millipore UFC910024) and the unlinked probe DNA was existed in effluent. The amount of unlinked biotin-DNA was quantified by Eppendorf BioPhotometer plus for the binding kinetics analysis.

### qIPCR mix and running program

ABA sample (1 μL), probe complex (1 μL) and ddH_2_O (8 μL) were added into McAb coated PCR plate and incubated for 2 h at 4 °C. Then, the solution in coated PCR plate-wells were removed and the plate-wells were washed three times with PBS. Finally, a 10 μL reaction solution was made. Specific primers (5′-CCGGTTCCCAACGATCAAG-3′ and 5′-AACCGCTTTTTTGCACAACAT-3′, each 1 μL), a certain volume of components of real-time PCR kit and ddH_2_O (up to 10 μL) were piped into PCR plate. Real-time PCR was performed on StrataGene Mx3000p Real-time PCR system (USA). The following programs were employed: pre-denaturing for 10 min at 95 °C, then amplifying for 40 cycles including denaturing for 30 s at 95 °C, annealing for 30 s at 56 °C and extending for 10 s at 72 °C.

### Plant materials and sampling

*Arabidopsis* and rice (*Oryza sativa*) seeds were surface sterilized by 70% alcohol and 5% NaClO (v/v), washed 3 times, and placed in the dark for 48 h at 4 °C to synchronize germination. The seedlings were grown in Murashige and Skoog (MS) medium [35] solidified with 0.4% (w/v) phytagel (Sigma-Aldrich Co. LLC, USA). Then both seedlings were placed vertically in a growth chamber under 16 h of light and 8 h of dark at 22 °C and 32 °C respectively. About 100 mg fresh tissues of rice and *Arabidopsis* seedlings were collected in 2 mL centrifuge tube and were immediately frozen in liquid nitrogen before storage in − 86 °C ultra-low temperature freezer. Additionally, to collect *Arabidopsis* stem and flower, 10-day old seedlings were planted into pots and growth in green house under 16 h of light and 8 h of dark at 22 °C to mature stage. Sampling was identical to that of seedlings.

### ABA extraction and determination through LC–MS/MS

Plant tissue was ground in liquid nitrogen and then 1 mL 80% methanol was used to extract ABA for 4 h in dark at 4 °C. Centrifugation was performed to remove solid impurities at 15,000 g for 10 min. Dried extract was dissolved in 200 µL of sodium phosphate solution (0.1 mol L21, pH 7.8). This crude extract can be directly used for ABA quantification through qIPCR. For liquid chromatography-tandem mass spectrometry (8030 plus; Shimadzu) analysis, the crude extract was eluted through a Sep-Pak C18 cartridge (Waters) with 1.5 mL of 80% methanol. The eluate was vacuumed to dryness again and dissolved in 100 µL of 10% methanol; 5 µL of the purified sample solution was then injected into the liquid chromatography-tandem mass spectrometry system.

Liquid chromatography was performed using a 2-mm i.d. 375-mm Shim-pack XR ODS I column (2.2 µm; Shimadzu) under a column temperature of 40 °C. The mobile phase comprising solvent A (0.02% [v/v] aqueous acetic acid) and solvent B (100% [v/v] methanol) was employed in a gradient mode (time/A concentration/B concentration [min/%/%]: 0/90/10, 5/10/90, 6/10/90, and 6.1/80/20) at an eluent flow rate of 0.3 mL per min. The mass system was set to multiple reaction monitoring mode using electrospray ionization for different hormones. Negative ion mode was used. Other operational conditions, including nebulizing gas flow, drying gas flow, desolvation temperature, and heat block temperature, were also optimized using standards. Deuterium-labeled ABA (Olchemim) were used as internal standards. Collision energy of 216 eV and mass-to-charge ratio (*m*/*z*) of 263.2 were employed [36].

## Results

### Strategy of the ABA qIPCR

**Fig. 1:**
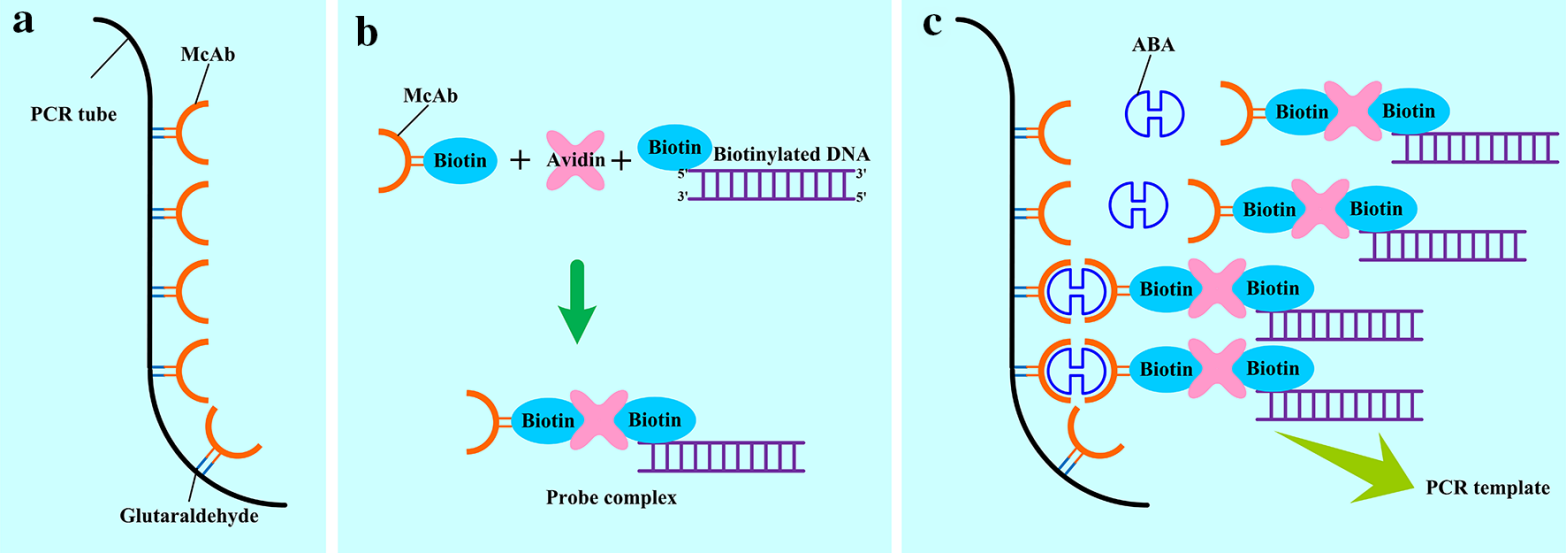
Operational scheme of qIPCR. **a** McAb is more efficiently coated on the inner surface of PCR tube after pretreating with glutaraldehyde; **b** probe complex is prepared in advance through linkage of streptavidin, biotin-McAb and biotin-DNA. The optimal molar mass ratio is 1:1:1 for biotin-McAb, avidin and biotin-DNA; **c** ABA sample and prepared probe complex are added into the PCR tube coated with McAb to crosslink. The trapped DNA represents the amount in ABA sample after washing PCR tube to remove the excessive probe complex. The trapped DNA can be quantified by RT-PCR

Binding affinity and specificity between analyte and its recognition factor are the key features for the accurate determination in IPCR. To obtain better binding specificity and higher sensitivity, ABA monoclonal antibody (McAb) was used in this study as the recognition factor. Additionally, we prepared the optimized probe complex solution containing biotinylated ABA McAb and probe DNA, and their crosslinking agent avidin beforehand,thus PCR mix in 96-well plate was rapidly prepared. The operational scheme of qIPCR was shown in Fig. 1: (1) through crosslinking reaction, ABA McAb was immobilized on the inner surface of PCR plate which was pretreated with glutaraldehyde (Fig. 1a). (2) Pre-preparing probe complex through avidin linking biotinylated ABA McAb and biotinylated probe DNA (Fig. 1b). (3) Adding probe complex and proper ABA sample into antibody coated tube for immuno-reaction, then washing the tube to remove excessive probe complex (Fig. 1c). (4) Making PCR mix and running the RT-PCR program.

### Optimized ABA antibody fixation

**Fig. 2:**
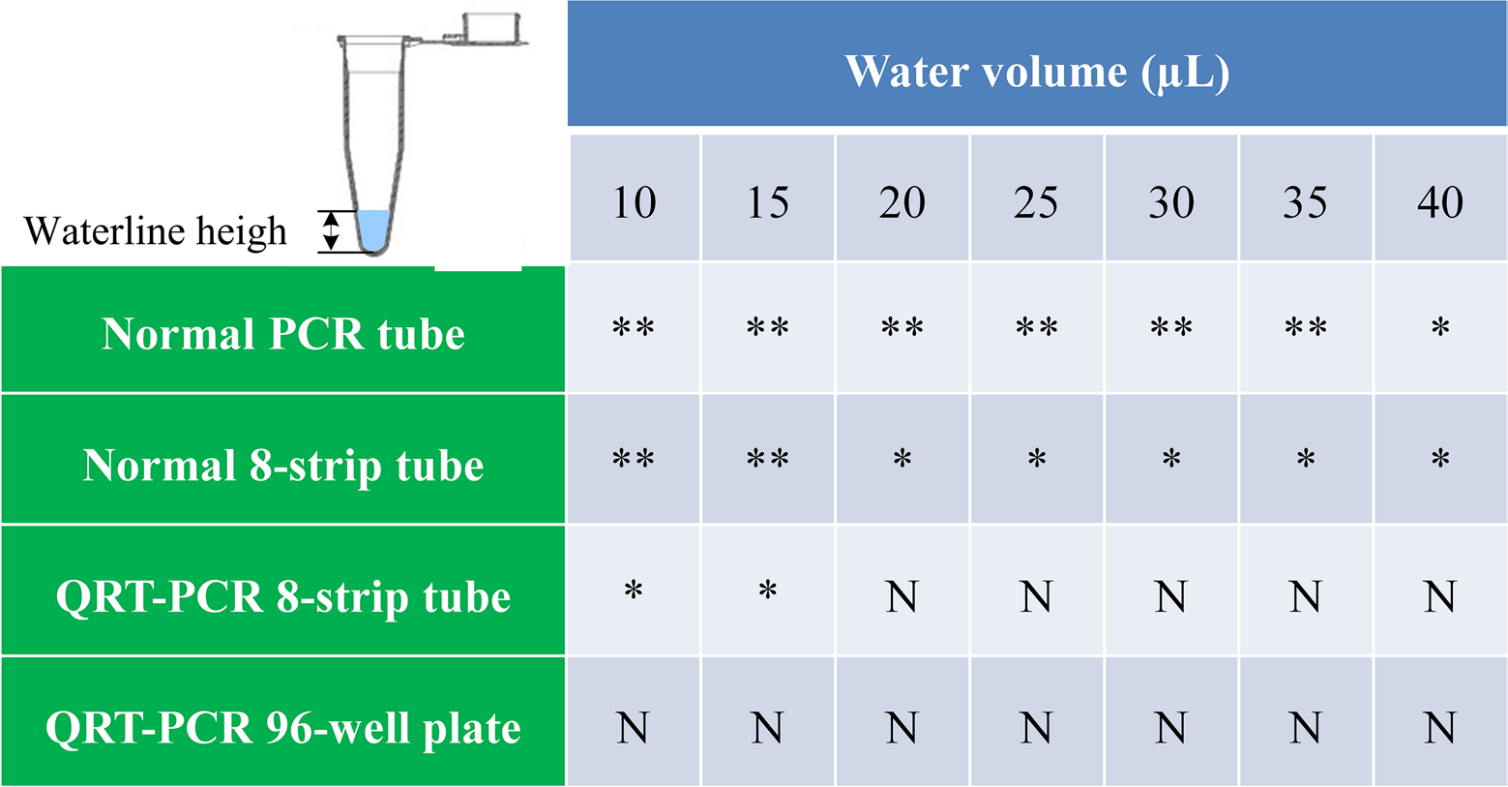
Homogeneity analysis of different type of PCR tube/plate. Water (from 10 to 40 µL) was pipetted into polypropylene (PP) PCR tubes including normal PCR tube, normal 8-strip tube, QRT-PCR tube and 96-well QRT-PCR plate respectively. The waterline height was measured by vernier caliper. Homogeneity of a certain type of PCR tube was determined through comparing the waterline height difference. The results represented the waterline height difference of same type PCR tubes when adding a certain volume of water. In all cases three independent biological replicates were analyzed. *N* not significant. *The difference was significant (p > 0.05). **The difference was extremely significant (p < 0.01)

PCR is an extreme signal enhancing method for DNA through exponential growth of products. In quantitative real time PCR, the amount of PCR products directly corresponds to the number of initial DNA template. For qIPCR, the consistency and stability of solidified probe DNA (that is DNA template) on the surface of PCR tube is a decisive factor for accurate quantification of analyte before performing PCR program. Therefore, high-homogeneity PCR plate, high-performance crosslinking agent and proper wash buffer were seriously considered before ABA McAb coating. To confirm high-homogeneity of the IPCR tube, we added certain volume of water in different type of polypropylene (PP) PCR tube and observed the waterline height to verify the homogeneity of PCR tube (Fig. 2). As shown in Fig. 2, ordinary PCR tube including normal PCR tube and 8-strip tube should be rejected to apply in IPCR because of higher inner surface deviation. Fortunately, most commercial 96/384-well QRT-PCR plates are homogeneous enough for high throughput quantification of analytes by qIPCR.

**Fig. 3:**
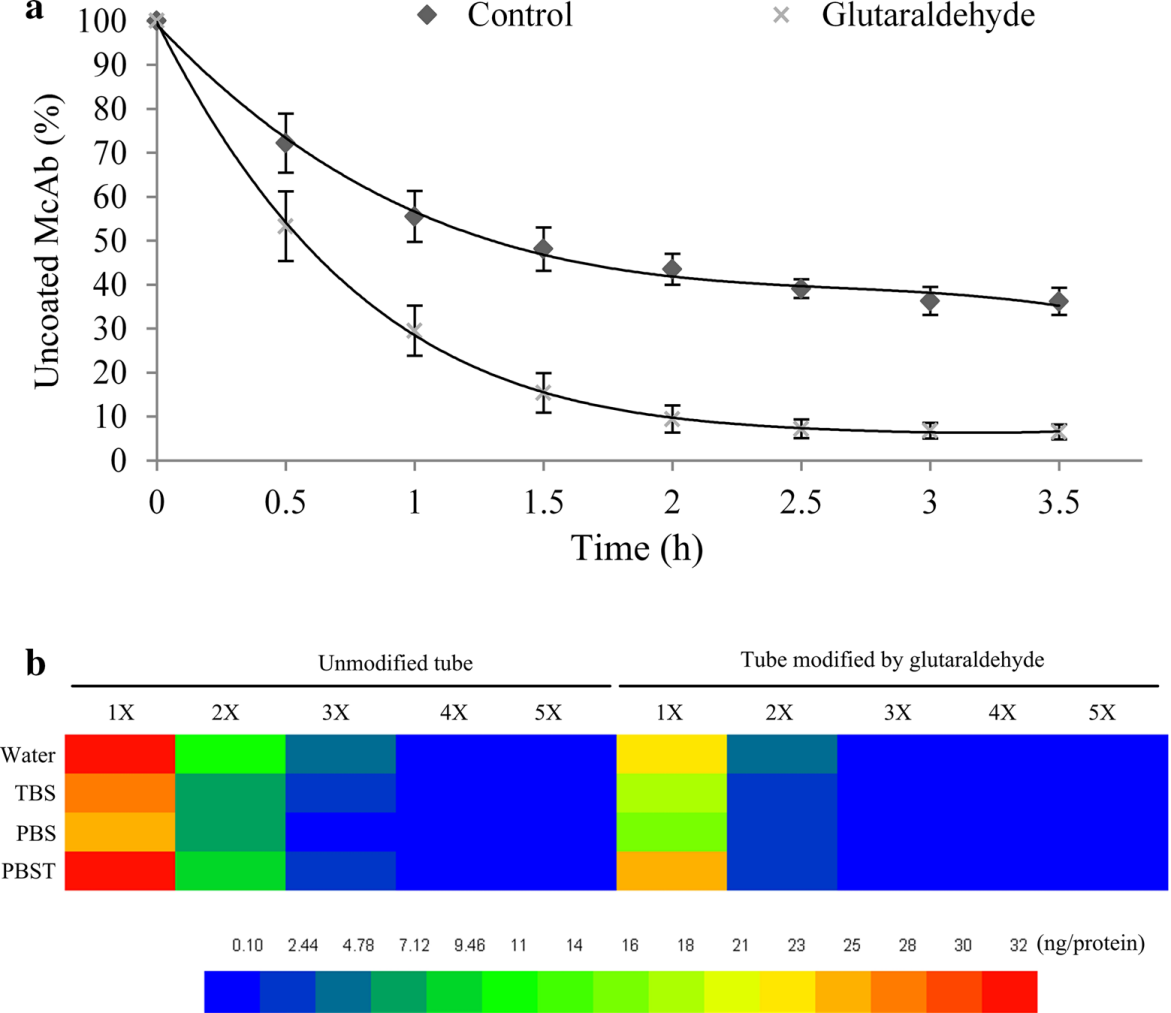
Effect of pretreatment with glutaraldehyde on the coating efficiency of ABA McAb. **a** Uncoated ABA McAb in wash buffer along with the coated time. Data represent the means and SD of three independent biological replicates (n = 5). **b** The binding stability analysis of ABA McAb coated (400 ng) on PCR tube in TBS (tris-buffered saline, 7.5), PBS (phosphate-buffered saline, 7.4), PBST (phosphate-buffered saline plus Tween, 7.5) and Milli Q water. X represents the number of wash times. The color meaning is the protein amount (ng) in washing buffer. Data represent the means of three independent biological replicates (n = 3)

Glutaraldehyde is currently used as an effective cross-linker in generating chemically, biologically and thermally stable cross-links with hydroxyl organics. Additionally, the superfluous glutaraldehyde can be easily removed by rinsing with normal mild saline (such as PBS, TBS, PBST and even hyperpure water) or quenching with organic solvent (such as methanol, chloroform and ether) since it shows higher solubility in both organic and aqueous phases [37, 38]. Moreover, glutaraldehyde can be employed in pretreating polyethylene or polypropylene tube to enhance binding characteristics with hydroxyl organics [30, 39]. Frequently-used PCR tube is made of polyethylene or polypropylene which owns lower binding capability and stability to proteins/antibodies comparing with polystyrene ELISA microplate. Since the PCR tube is an important restriction factor to affect sensitivity and repeatability of IPCR, we applied glutaraldehyde to pretreat PCR tube and performed binding dynamics analysis through detecting concentration of uncoated antibodies in removed solution from PCR tube at different time intervals. The results showed that PCR tube modified by glutaraldehyde rapidly reached saturation stage in 2 h. The antibody binding efficiency of glutaraldehyde treated tube was significantly higher than that of the untreated (Fig. 3a).

IPCR requires the antibody to stably bind to the solid phase surface. To test the binding stability of PCR tube and anti-ABA McAb, we detected the protein amount in washing buffer, including TBS (tris-buffered saline, 7.5), PBS (phosphate-buffered saline, 7.4), PBST (phosphate-buffered saline plus Tween, 7.5) and Milli Q water. The total protein content in whole volume washing buffer from antibody coated tube modified by glutaraldehyde was significantly lower than that from untreated tube, regardless the type of washing buffer used (Fig. 3b). This indicated that glutaraldehyde is an ideal stabilizer, thus pretreatment of PCR tube with glutaraldehyde before antibody coating was a recommended measure in qIPCR. Regarding the washing capability of different mild buffers, we found that more antibodies were detected in first two washing times using Milli Q water and PBST (Fig. 3b). We speculated that non-ionization water and Tween could elute part of bound McAb from coated PCR tube. TBS and PBS had no significant difference in washing uncoated antibody (Fig. 3b).

### Binding saturation analysis of fixed ABA McAb and antigen

**Fig. 4:**
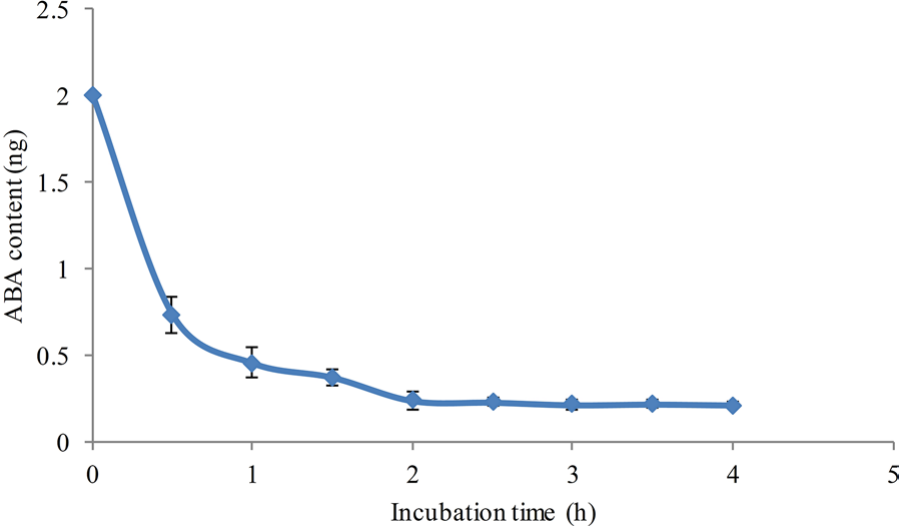
Binding saturation analysis between ABA and ABA McAb. ABA McAb (400 ng) was coated on inner PCR tube, then 2 ng ABA in 20 µL PBS was added into PCR tube and the mixture was incubated at 4 °C in dark. The solution in PCR tube was removed at half hour interval and ABA concentration was determined by LC–MS/MS. Data represent the means and SD of three independent biological replicates (n = 8)

In IPCR, unbounded analyte is removed along with washing buffer. Therefore, too much analyte in sample would lead to lower recovery and accuracy. The dose of analyte must not exceed the saturation and the upper limit of added ABA needs to be screened beforehand in qIPCR system. We analyzed the binding saturation between ABA McAb (about 400 ng) and ABA. Firstly, the PCR tube coated with McAb was prepared. Then after, 2 ng ABA in 20 µL PBS was added into PCR tube and the mixture was incubated at 4 °C in dark. The standard ABA solution was removed at half hour interval and ABA concentration in removed solution was determined by LC–MS/MS. The results showed that McAb quickly reacted with its antigen ABA within 2 h (Fig. 4).

### Binding kinetics of biotinylated ABA McAb and biotinylated probe DNA

**Fig. 5:**
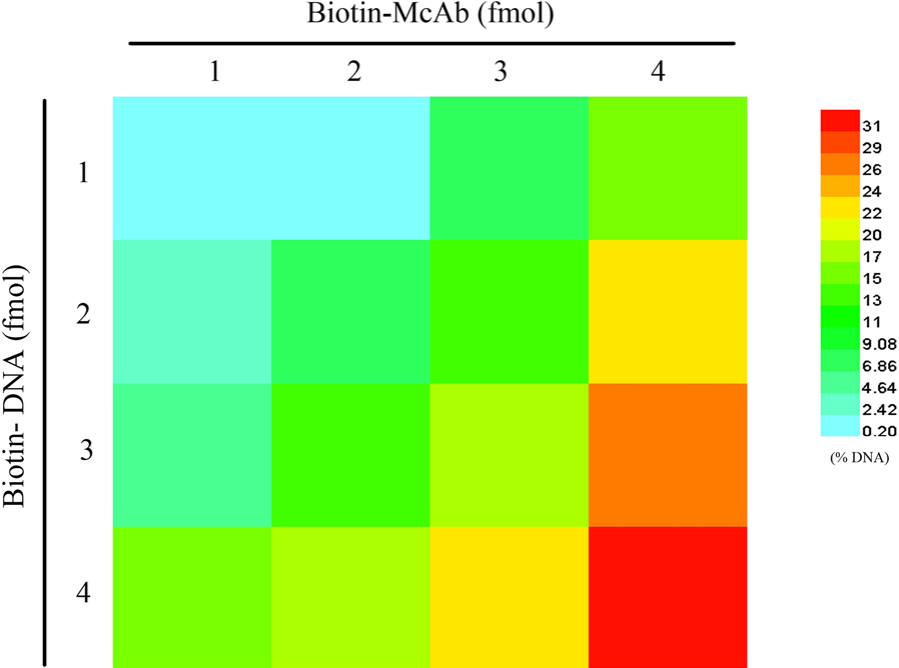
Binding kinetics of biotin-ABA McAb and biotin-DNA with avidin (1 fmol). The color meaning is percentage of unbound biotin-DNA in effluent after ultrafiltration through 100 kD ultra-filter tube (EMD Millipore UFC910024). Data represent the means of three independent biological replicates (n = 8)

To improve the stability of probe complex, we employed the biotin–avidin system. ABA McAb and probe DNA were beforehand biotinylated and then avidin was used as their cross-linker. Avidin contains four duplicate subunits and is a tetravalent binding glycoprotein for biotin to some extent. Competition exists between biotinylated ABA McAb and biotinylated probe DNA for binding site. To reveal the binding kinetics of biotin-McAb and biotin-DNA with avidin, we screened the molar mass ratio of biotin-McAb and biotin-DNA in the probe complex. In this test, the reaction solution contains one fmol avidin and different amounts of biotin-McAb and biotin-DNA (Fig. 5). The ultrafiltration was applied to separate unbound biotin-DNA and the DNA amount in outflow was determined. The results suggested that fewer biotin-McAb and biotin-DNA were preferred for unsaturated and stable probe complex. The optimal molar mass ratio was 1:1:1 for biotin-McAb, avidin and biotin-DNA (Fig. 5). Moreover, ultrafiltration was also employed to purify the probe complex.

### Standard curve and recovery analysis

**Fig. 6:**
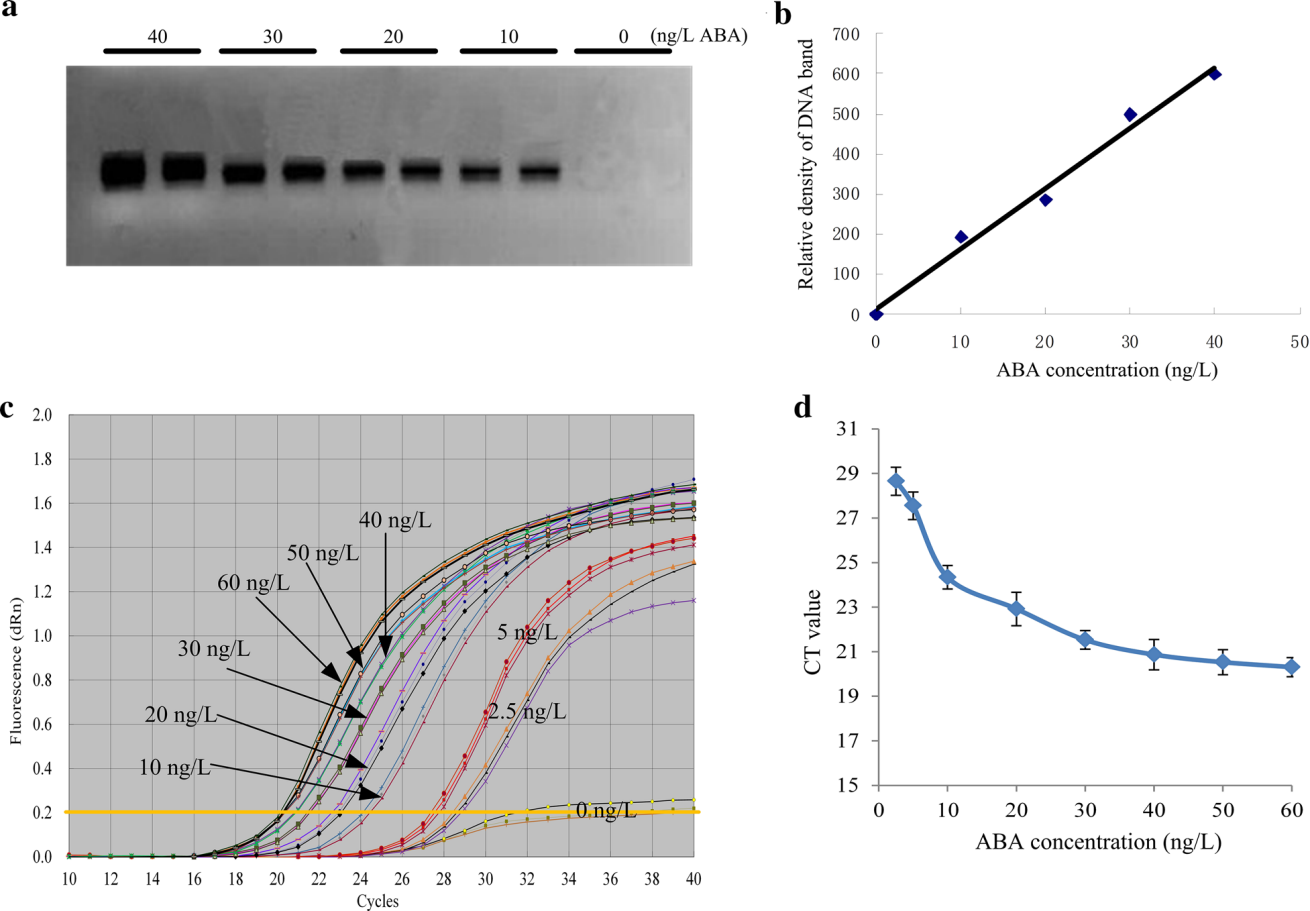
Amplification analysis of a serial concentrations of ABA by qIPCR. **a** qIPCR was performed in QRT-PCR tubes. The PCR products were exhibited in agarose gel; **b** the density values of DNA bands in agarose gel were estimated by *Image J* software (https://imagej.nih.gov/ij/index.html); **c** amplification curve of qIPCR in the exist of 0–60 ng/L ABA. SYBR green was used to quantify the DNA in 96-well QRT-PCR plate on StrataGene Mx3000p Real-time PCR system (USA); **d** relationship between standard ABA concentrations and CT values. It was linear in the range of 10 ng/L and 40 ng/L. In all cases three independent biological replicates were analyzed

Firstly, we screened the specificity of IPCR. The PCR products were exhibited in 2% agarose gel and no nonspecific band was found (Fig. 6a). Then, we calculated the relative densities of DNA bands through *Image J* software (https://imagej.nih.gov/ij/index.html). The concentration of ABA vs DNA density showed better linear relationship and the equation was y = 15.04x + 14.2 with the determinant coefficient (R^2^) of 0.9863 (Fig. 6b). Secondly, the real-time IPCR was performed on QRT PCR instrument through SYBR green method. The amplification curves (Fig. 6c) showed that this method could be applied in ABA quantification. The CT (cycle threshold) value would increase along with the decrease of the concentration of ABA (Fig. 6c). Subsequently, a standard curve was obtained using the relationship between the concentration of ABA standard solution and the average CT value in which ABA was linear in the range of 10 ng/L and 40 ng/L (Fig. 6d). The linear regression equation was y = − 0.118x + 25.365 with the determinant coefficient (R^2^) of 0.9763. The limit of detection (LOD) was 10 ng/L, which was converted as 2.5 fg ABA in one PCR reaction when utilizing 2.5 ng/L standard ABA solution. The repeatability and sensitivity of qIPCR method was very close to LC–MS/MS. In addition, the average recovery of ABA in the qIPCR method was 96.9%, similar to that of LC–MS/MS method of 98.1%.

### High throughput analysis of ABA in plant samples

qIPCR offers a rapid and high throughput method to quantify analyte but is prone to interference in a number of ways. Thus, it is advisable to validate for a particular tissue by comparing to the qIPCR data with those obtained by a main stream technique with proven veracity. For this purpose, the qIPCR was validated in comparison with LC–MS/MS using deuterium-labelled ABA as an internal standard in this paper. To further test the repeatability of qIPCR, we determined ABA content in eight different tissues from *Arabidopsis* and rice by 96-well plates. Comparing the ABA contents in plant tissues determined by qIPCR and LC–MS/MS, the differences between two methods for both the detected ABA concentration and repeatability were not significant (Table 1).

**Table 1:** ABA content in different tissues of *Arabidopsis* and rice (ng/g, fresh weight)

Plant tissue	qIPCR		HPLC–MS/MS	
	Purified sample	Crude extract	Purified sample	Crude extract
*Arabidopsis* root	45.1 ± 1.5	46.1 ± 2.3	43.3 ± 0.7	–
*Arabidopsis* stem	41.2 ± 1.1	39.2 ± 1.7	42.4 ± 0.8	–
*Arabidopsis* leaf	35.4 ± 0.8	36.4 ± 2.1	34.8 ± 0.5	–
*Arabidopsis* flower	55.6 ± 1.3	58.6 ± 2.8	58.2 ± 0.9	–
*Arabidopsis* seed	289.2 ± 1.9	293.9 ± 4.3	276.5 ± 1.8	–
Rice seedling root	47.3 ± 0.8	46.3 ± 2.6	48.7 ± 1.1	–
Rice seedling leaf	32.9 ± 1.3	32.7 ± 1.8	33.5 ± 0.9	–
Mature rice seed	313.6 ± 3.1	317.4 ± 3.9	305 ± 2.2	–

The purified sample was collected through standard extraction and purification processes. The crude extract was dissolved in acetonitrile after extracting with 80% acetone, removing solid impurity and drying [36]. “–” Represented no data because crude extract was not allowed in HPLC–MS/MS. Data represent the means and SD of three independent biological replicates (n = 5)

To further analyze the anti-interference capability, we tried to detect ABA content in the purified sample and the crude extract from different plant tissues (Table 1). The results indicated that qIPCR method showed high specificity for ABA and can be employed to accurately analyze the ABA in crude extracts. Of course, for higher accuracy, the salt content in sample should be limited in acceptable range because excessive ions would affect the amplification rate of PCR.

## Discussion

The immuno-PCR (IPCR) method has the potential to quantify analytes at sub-femto gram levels through the efficient amplification of PCR. Theoretically, IPCR can be applied to detect all molecules, but in previous publications, it was intensively employed in the quantification of bio-macromolecules, such as protein, nucleic acid and antibody [40–44]. Additionally, past efforts and practices were mostly focused on developing and screening the interlinking methods between small organic molecules and macromolecules. Consequently, IPCR has never been applied in phytohormonal quantification up to now although phytohormonal research is a hotspot in the plant biology field. This study is the first approach of IPCR application in phytohormonal quantification. In the ABA qIPCR, the biotin–avidin system was employed to be the linkage of ABA and DNA. In detail, the McAb against ABA and probe DNA were biotinylated, and then cross-linked as the probe complex through streptavidin in a certain mixture ratio (Figs. 1, 5). This strategy solved the problem that phytohormones are difficult to directly react with bio-macromolecules in vitro. The pre-preparation of the probe complex was very helpful for the standard and high throughput operations in the followed QRT-PCR.

For IPCR, the key factors affecting the sensitivity and efficiency are the affinity and binding specificity between the support substrate surface and the analytes, or between different molecules. Nonspecific binding will lead to a certain level of DNA-tag amplification in samples (background level). In practice, the main aim of optimization of the IPCR method is to quickly obtain high level of amplification in the samples containing analyte comparing to the negative controls [43]. In detail, the choice of support substrate, including binding characteristics and conjugation methods, should be seriously considered. In this study, we confirmed the type of PCR tubes and the pretreatment method in order to obtain high antibody/protein affinity and amplification efficiency. 96-well microplates show higher affinity for proteins/antibodies and are widely used for ELISA. However, microplates are not suitable for running PCR program because of heat-instability in a PCR device. If using microplates, an additional step is that probe DNA needs to be detached from the antigen–antibody complex and to be transferred into another PCR tubes for amplification. Polypropylene PCR tubes were used for IPCR, but did not provide the needed binding characteristic for protein/antibody [45, 46]. Polycarbonate tube have improved protein-binding capacity and heat-stability, but experiments carried out in polycarbonate tube showed low amplification efficiency and detection sensitivity because of non-uniform distribution of heat in wells during PCR [47, 48] although the problem was partly solved through increasing denaturation temperature and elongation time [49, 50]. In this study, we screened high quality polypropylene PCR tube and pretreated with 0.8% glutaraldehyde. The treated polypropylene PCR tube acquired significant improvement on the antibody-coating capability (Fig. 3a).

Plant extracts are extremely complex, but phytohormones are present at trace amounts in plants, usually at the level of 0.1–50 ng/g fresh weight. An ideal analytical method needs to be highly selective and sensitive in quantifying phytohormones. In the past decades, mass spectrometry (MS) has undergone spectacular development and become the main stream method in phytohormonal analysis for its extra sensitivity. Among them, LC–MS/MS is a powerful tool, but involves extra high costs, skilled operation and time consuming multi-step sample preparation. In this study, an easy-to-follow quantification method with extra sensitivity was developed to quantify ABA. In the ABA qIPCR, the limit of detection (LOD) reached 2.5 pg and it showed good reproducibility at femtogram level of ABA. The sensitivity and recovery were very close to that of LC–MS/MS. Moreover, the ABA qIPCR offered a sensitive and high throughput new method to quantify ABA in plant sample. Comparing with LC–MS/MS, an important improvement was that qIPCR can be used to analyze ABA in crude extract for plant samples. To fully exploit the advantages and potential of IPCR, further optimizations are still needed in protein coating, sampling and PCR condition. Under optimized conditions, qIPCR is expected to be applied in phytohormonal quantification at sub-femto gram level.

## Conclusion

The ABA qIPCR is an easy-to-follow and extra sensitive method for phytohormonal quantification in trace plant sample. It can be applied in accurate detection of the ABA in crude extract for plant sample with high specificity and repeatability. The sensitivity and recovery of the qIPCR was very close to that of the widely recognized quantification method of LC–MS/MS.
